# Causes, temporal trends, and the effects of urbanization on admissions of wild raptors to rehabilitation centers in England and Wales

**DOI:** 10.1002/ece3.8856

**Published:** 2022-04-20

**Authors:** Connor T. Panter, Simon Allen, Nikki Backhouse, Elizabeth Mullineaux, Carole‐Ann Rose, Arjun Amar

**Affiliations:** ^1^ School of Geography University of Nottingham Nottingham UK; ^2^ Gower Bird Hospital Swansea UK; ^3^ Cuan Wildlife Rescue The Signals Shropshire UK; ^4^ Secret World Wildlife Rescue Somerset UK; ^5^ Wild Wings Birds of Prey Warrington UK; ^6^ Fitzpatrick Institute of African Ornithology DSI‐NRF Centre of Excellence University of Cape Town Rondebosch South Africa

**Keywords:** birds of prey, conservation, morbidity, mortality, threats, wildlife rescue centers

## Abstract

Data from wildlife rehabilitation centers (WRCs) can provide on‐the‐ground records of causes of raptor morbidity and mortality, allowing threat patterns to be explored throughout time and space. We provide an overview of native raptor admissions to four WRCs in England and Wales, quantifying the main causes of morbidity and mortality, trends over time, and associations between threats and urbanization between 2001 and 2019. Throughout the study period, 14 raptor species were admitted totalling 3305 admission records. The Common Buzzard (*Buteo buteo*; 31%) and Tawny Owl (*Strix aluco*; 29%) were most numerous. Relative to the proportion of breeding individuals in Britain and Ireland, Peregrine Falcons (*Falco peregrinus*), Little Owls (*Athene noctua*), and Western Barn Owls (*Tyto alba*) were over‐represented in the admissions data by 103%, 73%, and 69%, respectively. Contrastingly Northern Long‐eared Owls (*Asio otus*), Western Marsh Harriers (*Circus aeruginosus*), and Merlin (*Falco columbarius*) were under‐represented by 187%, 163%, and 126%, respectively. Across all species, vehicle collisions were the most frequent anthropogenic admission cause (22%), and orphaned young birds (10%) were most frequent natural cause. Mortality rate was highest for infection/parasite admissions (90%), whereas orphaned birds experienced lowest mortality rates (16%). For one WRC, there was a decline in admissions over the study period. Red Kite (*Milvus milvus*) admissions increased over time, whereas Common Buzzard and Common Kestrel admissions declined. There were significant declines in the relative proportion of persecution and metabolic admissions and an increase in orphaned birds. Urban areas were positively associated with persecution, building collisions, and unknown trauma admissions, whereas vehicle collisions were associated with more rural areas. Many threats persist for raptors in England and Wales, however, have not changed substantially over the past two decades. Threats associated with urban areas, such as building collisions, may increase over time in line with human population growth and subsequent urban expansion.

## Graphical abstract

We provide an overview of native raptor admissions to four WRCs in England and Wales. Vehicle collisions and orphaned birds were the most frequently identified causes of admission. Urban areas were positively associated with persecution, building collisions, and unknown trauma, whereas vehicle collisions were associated with more rural areas.

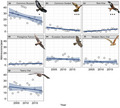

## 1 INTRODUCTION

Diurnal and nocturnal raptors are frequently used as ecological indicators due to their high positions within trophic networks (Buechley et al., 2019). Raptor species face a number of threats from anthropogenic activities such as direct and indirect poisoning (Garvin et al., 2020; Hughes et al., 2013), electrocution on powerlines (Lehman et al., 2007), road collisions (Gagné et al., 2015), and human persecution (Murgatroyd et al., 2019; Panter et al., 2021; Smart et al., 2010). For effective conservation programs, the key detrimental impacts of anthropogenic activities need to be identified and evidenced‐based conservation measures implemented to alleviate these threats (Hernandez et al., 2018; Holmes et al., 1993; Richardson & Miller, 1997).

Several methods have been applied to quantify the effects of anthropogenic activities on raptors. Such approaches include screening for organic pollutants and contaminants (Chen et al., 2010; López et al., 2001), monitoring the dynamics of the illicit wildlife trade (Panter & White, 2020), analysis of powerline collision data (Bevanger, 1998; Kolnegari et al., 2020), monitoring via remote tracking devices (Kendall & Virani, 2012; McIntyre, 2012; Panter et al., 2020, 2021), and analysis of wildlife rehabilitation admission data (see Al Zoubi et al., 2020; Fix & Barrows, 1990; Komnenou et al., 2005; Molina‐López et al., 2011; Molina‐López & Darwich, 2011; Morishita et al., 1998; Rodríguez et al., 2010; Thompson et al., 2013; Wendell et al., 2002).

Raptor data from wildlife rehabilitation centers provide on‐the‐ground records of causes of morbidity and mortality and have been used to evaluate the health status of wild populations (Morishita et al., 1998; Wendell et al., 2002) and to explore trends in anthropogenic threats over time (Molina‐López et al., 2011; Thompson et al., 2013). Rehabilitation and subsequent release of individuals back into the wild can help to buffer the negative effects of anthropogenic activities, especially for species of conservation concern (Dessalvi et al., 2021; Hernandez et al., 2018; Montesdeoca, Calabuig, Corbera, Cooper, et al., 2017; Mullineaux, 2014; Romero et al., 2019; Thomson et al., 2020).

While several previous studies have explored morbidity and mortality of raptors based on admission data to rehabilitation centers, most of these were based on data from a single center, limiting their ability to explore patterns in admission causes over larger spatial scales. To our knowledge, no studies have attempted to explore whether causes of morbidity or mortality differ depending on environmental features and very few have been conducted in the United Kingdom. For example, Kelly and Bland (2006) analyzed admissions, diagnoses, and outcomes of raptors admitted to a center in England, focusing on a single species—the Eurasian Sparrowhawk (*Accipiter nisus*).

In this study, we compile and analyze raptor admission data from four wildlife rehabilitation centers in western/south‐western England and Wales. Firstly, we provide an overview of raptor admissions over a 19‐year period (2001–2019), quantifying the most frequently admitted species and the main causes. We then explore whether a number of commonly admitted species and the types (anthropogenic vs natural) or causes of admission have changed over time for one rehabilitation center, for which we had the longest run of data. Over the study period, urban cover in England and Wales has increased (Office for National Statistics, 2021). Therefore, we predict an increase in anthropogenic admissions as a result of increasing human population growth and urban expansion over time (Seto et al., 2012). Certain threats may also have changed over time; for example, over the study period, the number of vehicles in England and Wales has increased (Department of Transport, 2020), and subsequent raptor‐vehicle collisions may have also increased over time. Finally, we expect that causes of admission will vary depending on the level of urbanization. For example, we might expect that urbanization increases the probability of admissions due to building or vehicle collisions in line with previous findings (Garcês et al., 2020; Loss et al., 2014). Therefore, we explore whether the level of urbanization (where the individual birds were found) is associated with higher probabilities of certain admission causes.

## 2 METHODS

### 2.1 Study area

**FIGURE 1:**
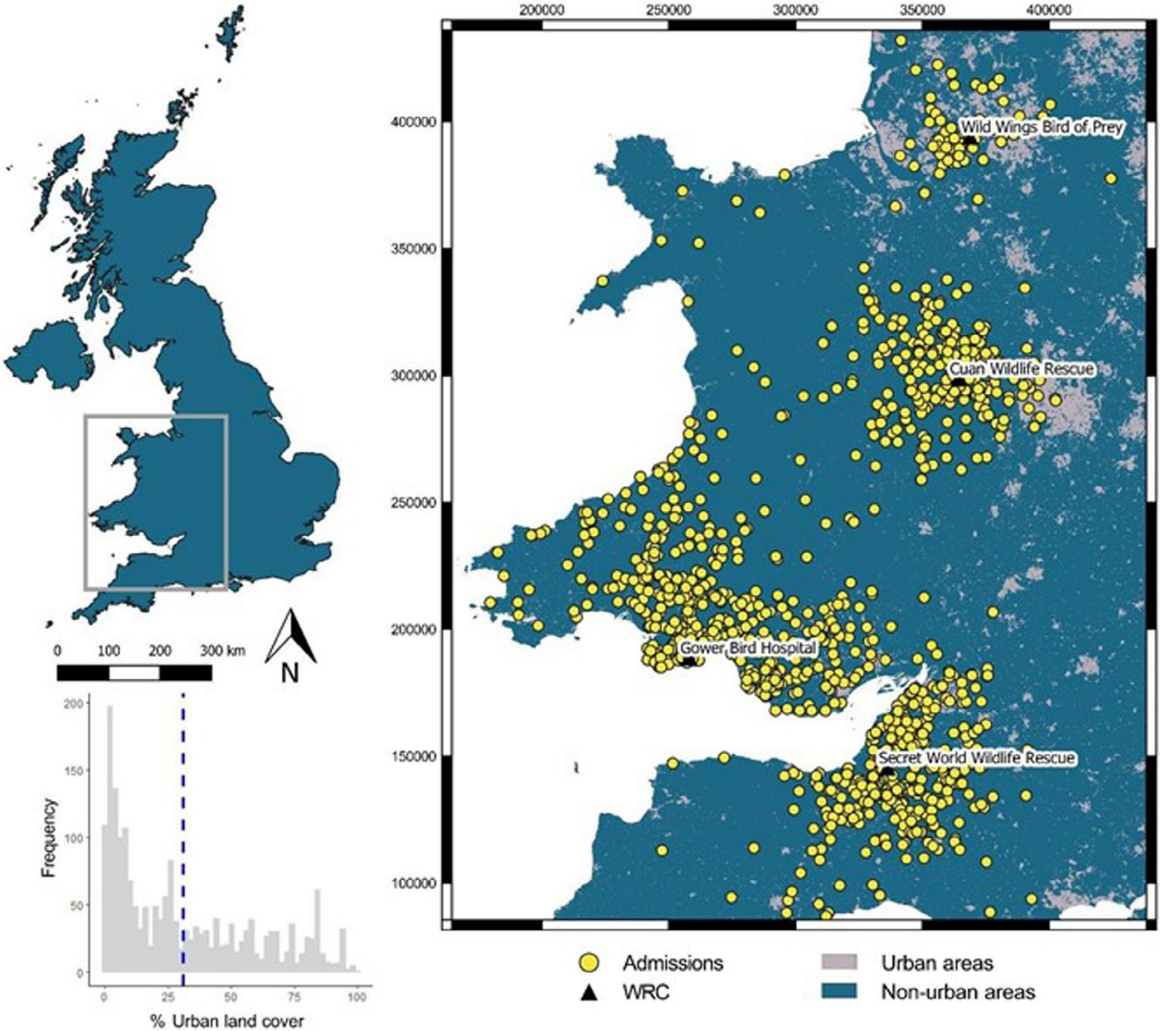
Spatial distribution for 14 species of diurnal and nocturnal raptors admitted to four wildlife rehabilitation centers (WRC) between 2001 and 2019 in England and Wales. Geo‐referenced admissions with 2‐km buffers (*N* = 1915) shown in relation to urban land cover. Histogram shows the frequency of urban land cover scores within each 2‐km buffer and the mean (31%) denoted by the blue dashed line. Map Coordinate Reference System: EPSG 27700 British National Grid

We collated admission records of native raptors admitted to wildlife rehabilitation centers (WRC) located within a study area totalling *c*. 46,000 km^2^ in south‐western Britain (Figure 1). The landscape within our study area is not only dominated by agriculture but also includes the major cities of Greater Manchester, Birmingham, Bristol, and Cardiff, which have populations of *c*. 2.8 million, 2.6 million, 690,000, and 495,000 people, respectively (United Nations, 2014). Our study area also includes the Brecon Beacons National Park, seven “Areas of Outstanding Natural Beauty” (AONB), and numerous “Sites of Special Scientific Interest” (SSSI) including the West Pennine Moors, Wyre Forest, and Quantock Hills.

### 2.2 Data collection

Wildlife rehabilitation centers were invited to participate in the study via email correspondence. Four WRC supplied data on raptor admissions to their centers: Cuan Wildlife Rescue (lat/long: 52.590, −2.573), Gower Bird Hospital (51.580, −4.099), Secret World Wildlife Rescue (51.206, −2.964), and Wild Wings Birds of Prey (53.444, −2.522). From their admission records, the following data were collected for each individual admitted: (1) species, (2) sex (male/female), (3) age (juvenile/adult; <1 calendar year/>1cy), (4) admission date, (5) cause of admission, (6) location of incident (at the finest spatial scale available), and (7) outcome (deceased/released/kept in captivity). These data spanned a 19‐year period from 21st January 2001 to 26th December 2019.

### 2.3 Classifying causes of morbidity and mortality

To increase comparability with other studies, the classification of admission causes followed categories previously defined by existing studies (see Molina‐López et al., 2011; Molina‐López & Darwich, 2011). Upon admission, birds were examined by trained wildlife carers and the admission notes associated with each record were used to assign each admission to the following “types” (“ANTHROPOGENIC,” “NATURAL,” and “UNKNOWN”) and more detailed “causes” (see Appendix S1 for an overview of all admission types, causes, codes, and pooled miscellaneous causes). When causes could not be ascertained, admission type was categorized as “UNKNOWN,” which included the causes: “undetermined” (reason unknown and no injury to bird) and “unknown trauma” (reason unknown but the bird was physically injured).

### 2.4 Landscape and demographic variables

To explore urbanization effects on types and causes of raptor admissions, we used only the geo‐referenced admissions (*N* = 1915). For these, we extracted land cover data and calculated the proportion of urban habitat within a 2‐km buffer. Land cover data were downloaded on 30^th^ April 2020 from the EDINA Environment Digimap Service (Land Cover Map, 2015; https://digimap.edina.ac.uk/). Land cover data were derived from the “LCM2015” data set in raster format at 25 m resolution, which closely aligned with the timescale of the majority of the admissions. All spatial data extraction was performed in QGIS 3.12.3 with the GRASS 7.4.1 extension (QGIS Development Team, 2019). We reclassified the land cover data using the *r*.*reclass* function and a new binary raster layer was created (1 = “urban” + “suburban” and 0 = all other land cover types). Summary statistics were then computed using the base function *Zonal Statistics* to calculate the percentage of urban cover within each 2‐km buffer.

### 2.5 Statistical analysis

All statistical analyses were performed in R version 3.6.3 (R Core Team, 2020). Data were analyzed using generalized linear models (GLMs) with either binomial (for binary models) or Poisson (for count data) distributions and the respective conical link functions (see Appendix S2 for a list of models). For binomial data, we fitted a two‐vector response variable using the cbind function. For Poisson GLMs where overdispersion was detected, we fitted the models with a quasi‐Poisson distribution.

We explored mortality (binary: 1 = bird died or was euthanised termed “deceased” and 0 = bird released or kept captive termed “not deceased”) as a response variable, with explanatory variables of either admission type or cause. We explored trends over time using only data from Gower Bird Hospital, as it was the only WRC with the longest run of data. Using these data, we fitted year as the explanatory variable and fitted a series of separate GLMs with the following response variables: (1) total count of admission each year, irrespective of cause and including unknown causes (Poisson model). (2) Total count of admission each year for the seven most frequently admitted species (with ≥30 admissions). (3) Relative proportion, per year, of admission causes (with ≥30 admissions). (4) Admission type, anthropogenic or natural (binomial model).

The effects of urbanization on types and causes of admissions were explored using a series of generalized linear mixed models (GLMMs) in the package “lme4” (Bates et al., 2015). For each admission, a binary metric was created (1 = matching admission type and 0 = no match) for each admission type (i.e., anthropogenic, natural, or unknown), or admission cause (where there were ≥30 admissions, i.e., vehicle collisions, trauma, undetermined, orphaned, building collisions, metabolic, infections/parasites and persecution). These models were then run with “binary admission type/cause” fitted as the response term and “% urban land cover” fitted as the explanatory term. We used binomial error distributions and “logit” link functions with “centreID” included as a random term to control for the lack of independence between admissions from the same center (Appendix S2).

We examined whether certain species were over‐ and under‐represented within our admissions data by calculating the percentage difference between the relative proportion of breeding individuals in Britain and Ireland, and the proportion of admitted individuals, per species, to each WRC. Breeding population data were derived from the British Trust for Ornithology's BirdFacts database (Robinson, 2005; https://www.bto.org/understanding‐birds/birdfacts).

## 3 RESULTS

Across the 19‐year study period, we recorded a total of 3305 admissions, comprising 14 species, (Table 1), with 1919 (58%) of admissions being diurnal species and 1386 (42%) being nocturnal species. The diurnal raptors comprised of nine species, the Common Buzzard (*Buteo buteo*) (*N* = 1035; 31%) was the most frequently admitted species, followed by the Eurasian Sparrowhawk (*Accipiter nisus*) (*N* = 457; 14%) and then the Common Kestrel (*Falco tinnunculus*) (*N* = 269; 8%). The Tawny Owl (*Strix aluco*) (*N* = 967; 29%) was the second most frequently admitted of all species and the most frequently admitted nocturnal species, followed by the Western Barn Owl (*Tyto alba*) (*N* = 283; 9%) and the Little Owl (*Athene noctua*) (*N* = 118; 4%).

**TABLE 1:** Demographics of diurnal and nocturnal raptor species admitted to four wildlife rehabilitation centers in England and Wales between 2001 and 2019

Species	Sex			Age			Total (%)
	Male (%/sp.)	Female (%/sp.)	Unknown (%/sp.)	Adult (%/sp.)	Juvenile (%/sp.)	Unknown (%/sp.)	
*Diurnal*							
Common Buzzard (*Buteo buteo*)	107 (10)	120 (12)	808 (78)	615 (59)	287 (28)	133 (13)	1035 (31)
Eurasian Sparrowhawk (*Accipiter nisus*)	92 (20)	129 (28)	236 (52)	240 (53)	158 (35)	59 (13)	457 (14)
Common Kestrel (*Falco tinnunculus*)	48 (18)	38 (14)	183 (68)	114 (42)	122 (45)	33 (12)	269 (8)
Peregrine Falcon (*Falco peregrinus*)	28 (33)	22 (26)	34 (40)	44 (52)	36 (43)	4 (5)	84 (3)
Red Kite (*Milvus milvus*)	3 (8)	4 (11)	29 (81)	27 (75)	9 (25)		36 (1)
Eurasian Hobby (*Falco subbuteo*)	1 (6)	2 (12)	14 (82)	12 (71)	1 (6)	4 (24)	17 (1)
Northern Goshawk (*Accipiter gentilis*)	5 (31)	6 (38)	5 (31)	3 (19)	13 (81)		16 (<1)
Merlin (*Falco columbarius*)	1 (25)	1 (25)	2 (50)	1 (25)	3 (75)		4 (<1)
Western Marsh Harrier (*Circus aeruginosus*)			1 (100)			1 (100)	1 (<1)
Total diurnal^a^	285 (15)	322 (17)	1312 (68)	1056 (55)	629 (33)	234 (12)	1919 (58)
*Noctural*							
Tawny Owl (*Strix aluco*)	35 (4)	21 (2)	911 (94)	474 (49)	359 (37)	134 (14)	967 (29)
Western Barn Owl (*Tyto alba*)	39 (14)	54 (19)	190 (67)	156 (55)	98 (35)	29 (10)	283 (9)
Little Owl (*Athene noctua*)	1 (1)	1 (1)	116 (98)	41 (35)	63 (53)	14 (12)	118 (4)
Short‐eared Owl (*Asio flammeus*)		3 (19)	13 (81)	12 (75)	3 (19)	1 (6)	16 (<1)
Northern Long‐eared Owl (*Asio otus*)			2 (100)	2 (100)			2 (<1)
Total nocturnal^a^	75 (5)	79 (6)	1232 (89)	685 (49)	523 (38)	178 (13)	1386 (42)
Total admissions	360 (11)	401 (12)	2544 (77)	1741 (53)	1152 (35)	412 (12)	3305 (100)

Note

Demographic proportions calculated per species, total calculated based on total number of admissions.

^a^ Proportions calculated using total diurnal and nocturnal values.

Only 761 (23%) admitted birds were successfully sexed, of these 47% were males and 53% were females. Age was determined for 2893 (88%) admissions with adults (>1cy) representing 60% and juveniles (<1cy) 40% of aged individuals (Table 1).

### 3.1 Admission types and causes

Unknown admission types were the most numerous comprising nearly half of all admissions (*n* = 1510; 46%), followed by anthropogenic (*N* = 1215; 37%) then natural admission types (*N* = 580; 17%; Table 2). Classifying admissions by the more detailed “causes” revealed 855 (26%) of all admissions were associated with “unknown trauma” (Table 2). The most frequent anthropogenic admission cause was “vehicle collisions” (*N* = 732; 22% of all admissions; 60% of anthropogenic admissions). For natural admissions, orphaned young birds were the most frequent cause (*N* = 315; 10% of all admissions, 54% of natural admissions; Table 2).

**TABLE 2:** Admission types and causes for 14 species of diurnal and nocturnal raptors, admitted to four wildlife rehabilitation centers in England and Wales between 2001 and 2019

Species admitted	Anthropogenic (%/sp.)							Natural (%/sp.)				Unknown (%/sp.)		Total (%)
	attack	build	elec	fence	habitat	pers	veh	infect	metab	orph	pred	trauma	undet	
*Diurnal*														
Common Buzzard (*Buteo buteo*)		26 (3)	10 (1)	12 (1)	3 (<1)	25 (2)	262 (25)	30 (3)	68 (7)	20 (2)	8 (1)	333 (32)	238 (23)	1035 (31)
Eurasian Sparrowhawk (*Accipiter nisus*)	19 (4)	105 (23)	1 (<1)	9 (2)		15 (3)	40 (9)	13 (3)	8 (2)	6 (1)	6 (1)	163 (36)	72 (16)	457 (14)
Common Kestrel (*Falco tinnunculus*)	1 (<1)	15 (6)	2 (1)	2 (1)	1 (<1)	1 (<1)	37 (14)	3 (1)	26 (10)	38 (14)		80 (30)	63 (23)	269 (8)
Peregrine Falcon (*Falco peregrinus*)		2 (2)		3 (4)		5 (6)	6 (7)	1 (1)	2 (2)	10 (12)	2 (2)	38 (45)	15 (18)	84 (3)
Red Kite (*Milvus milvus*)		5 (14)				1 (3)	9 (25)			2 (6)		4 (11)	15 (42)	36 (1)
Eurasian Hobby (*Falco subbuteo*)		3 (18)					4 (24)				1 (6)	5 (29)	4 (24)	17 (1)
Northern Goshawk (*Accipiter gentilis*)		3 (19)	1 (6)			1 (6)			1 (6)	1 (6)		7 (44)	2 (13)	16 (<1)
Merlin (*Falco columbarius*)		1 (25)										3 (75)		4 (<1)
Western Marsh Harrier (*Circus aeruginosus*)												1 (100)		1 (<1)
Total diurnal^a^	20 (1)	160 (8)	14 (1)	26 (1)	4 (<1)	48 (3)	358 (19)	47 (2)	105 (5)	77 (4)	17 (1)	634 (33)	409 (21)	1919 (58)
*Noctural*														
Tawny Owl (*Strix aluco*)	8 (1)	82 (8)		41 (4)	8 (1)	17 (2)	290 (30)	35 (4)	24 (2)	154 (16)	6 (1)	133 (14)	169 (17)	967 (29)
Western Barn Owl (*Tyto alba*)	3 (1)	9 (3)	1 (<1)	2 (1)	6 (2)	7 (2)	66 (23)	6 (2)	11 (4)	50 (18)	4 (1)	60 (21)	58 (20)	283 (9)
Little Owl (*Athene noctua*)	5 (4)	13 (11)			5 (4)	1 (1)	17 (14)		5 (4)	34 (29)	5 (4)	18 (15)	15 (13)	118 (4)
Short‐eared Owl (*Asio flammeus*)					1 (6)	1 (6)	1 (6)					9 (56)	4 (25)	16 (<1)
Northern Long‐eared Owl (*Asio otus*)						1 (50)						1 (50)		2 (<1)
Total nocturnal^a^	16 (1)	104 (8)	1 (<1)	43 (3)	20 (1)	27 (2)	374 (27)	41 (3)	40 (3)	238 (17)	15 (1)	221 (16)	246 (18)	1386 (42)
Total	36 (1)	264 (8)	15 (<1)	69 (2)	24 (<1)	75 (2)	732 (22)	88 (3)	145 (4)	315 (10)	32 (1)	855 (26)	655 (20)	3305 (100)

Note

Causes: “attack” = attacked by pet, “build” = building collisions, “elec” = electrocutions, “fence” = fencing/entanglements, “habitat” = habitat destruction, “pers” = persecutions, “veh” = vehicle collisions, “infect” = infection/parasites, “metab” = metabolic, “orph” = orphaned, “pred” = predation, “trauma” = unknown trauma, and “undet” = undetermined. See Table S1 for full‐cause descriptions.

^a^ Proportions calculated using total diurnal and nocturnal values.

**FIGURE 2:**
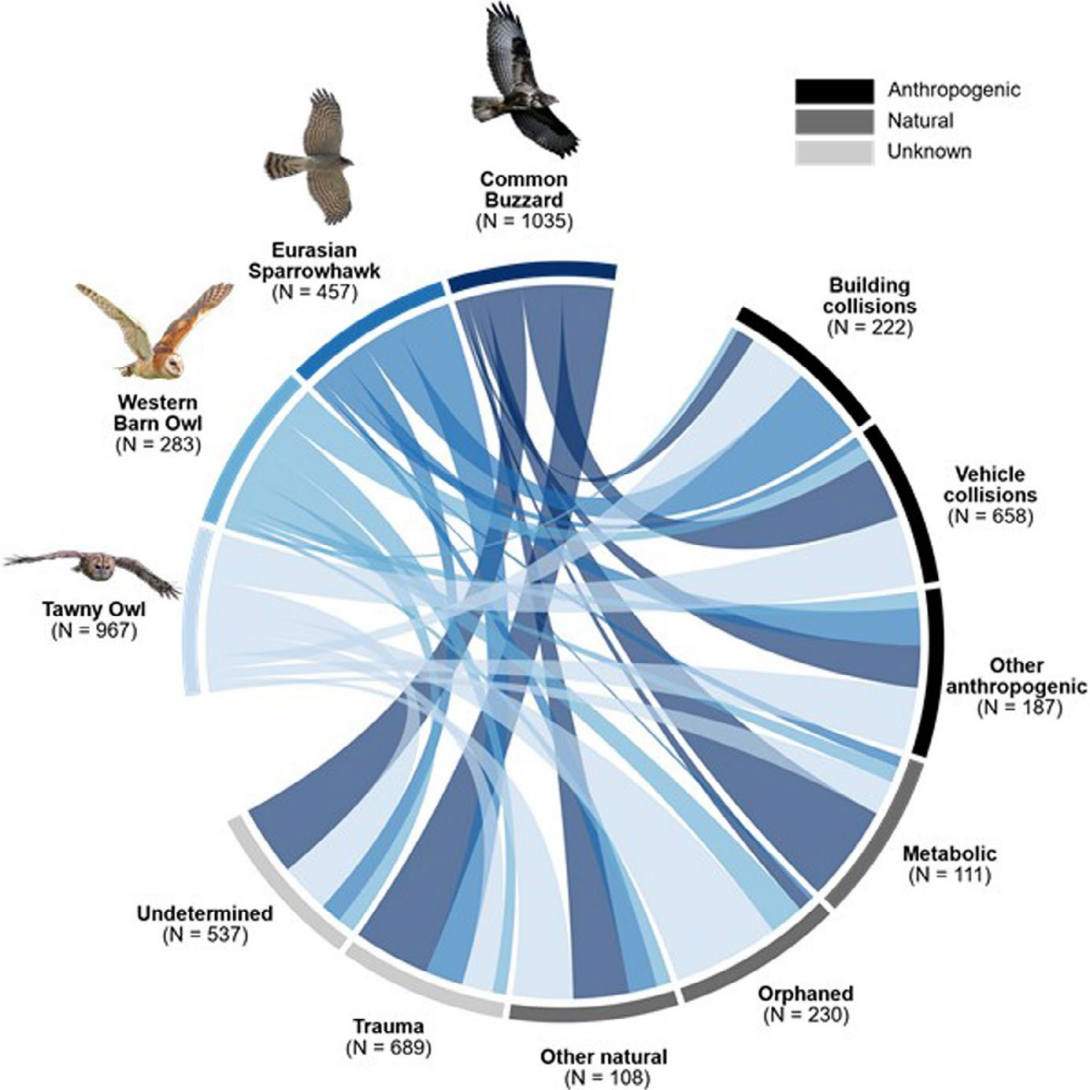
Admission causes for the top two most common diurnal and nocturnal raptor species admitted to four wildlife rehabilitation centers between 2001 and 2019 (*N* = 3011). Only the two most common admission causes per type (anthropogenic, natural, and unknown) shown, other causes pooled into respective categories: “Other anthropogenic” causes include “attacked” (*N* = 30), “fencing/entanglement” (*N* = 64), “electrocution” (*N* = 12), “habitat destruction” (*N* = 17), and “persecution” (*N* = 64). “Other natural” causes include “infection/parasites” (*N* = 84) and “predation” (*N* = 24)

When exploring only identified admission causes (excluding all unknown admission causes), vehicle collisions were the most common cause for five species including the Common Buzzard (56%; *N* = 262/464), Red Kite (*Milvus milvus*; 53%; 9/17), Eurasian Hobby (*Falco subbuteo*; 50%; 4/8), Tawny Owl (44%; 290/665), and Western Barn Owl (40%; 66/165) (Table 2). For the two most admitted diurnal species, the Common Buzzard and Eurasian Sparrowhawk, unknown trauma was the most common admission cause (Figure 2). Main admission causes for Tawny Owls were vehicle collisions and orphaned young birds, comprising 40% and 49% of admissions, respectively (Table 2; Figure 2).

Juvenile birds were approximately four times more likely to be admitted due to natural admissions than adults (430 vs. 112 admissions, respectively), and one and half times more likely to be admitted due to metabolic causes, e.g., emaciation or starvation, (79 vs. 54 admissions, respectively). Orphaned young birds totalled 10% (315) of all admissions and were the most frequent known admission cause for the Common Kestrel (14%; 38/269), Little Owl (29%; 34/118), and Peregrine Falcon (*Falco peregrinus*; 12%; 10/84) (Table 2).

### 3.2 Outcome of admissions

**FIGURE 3:**
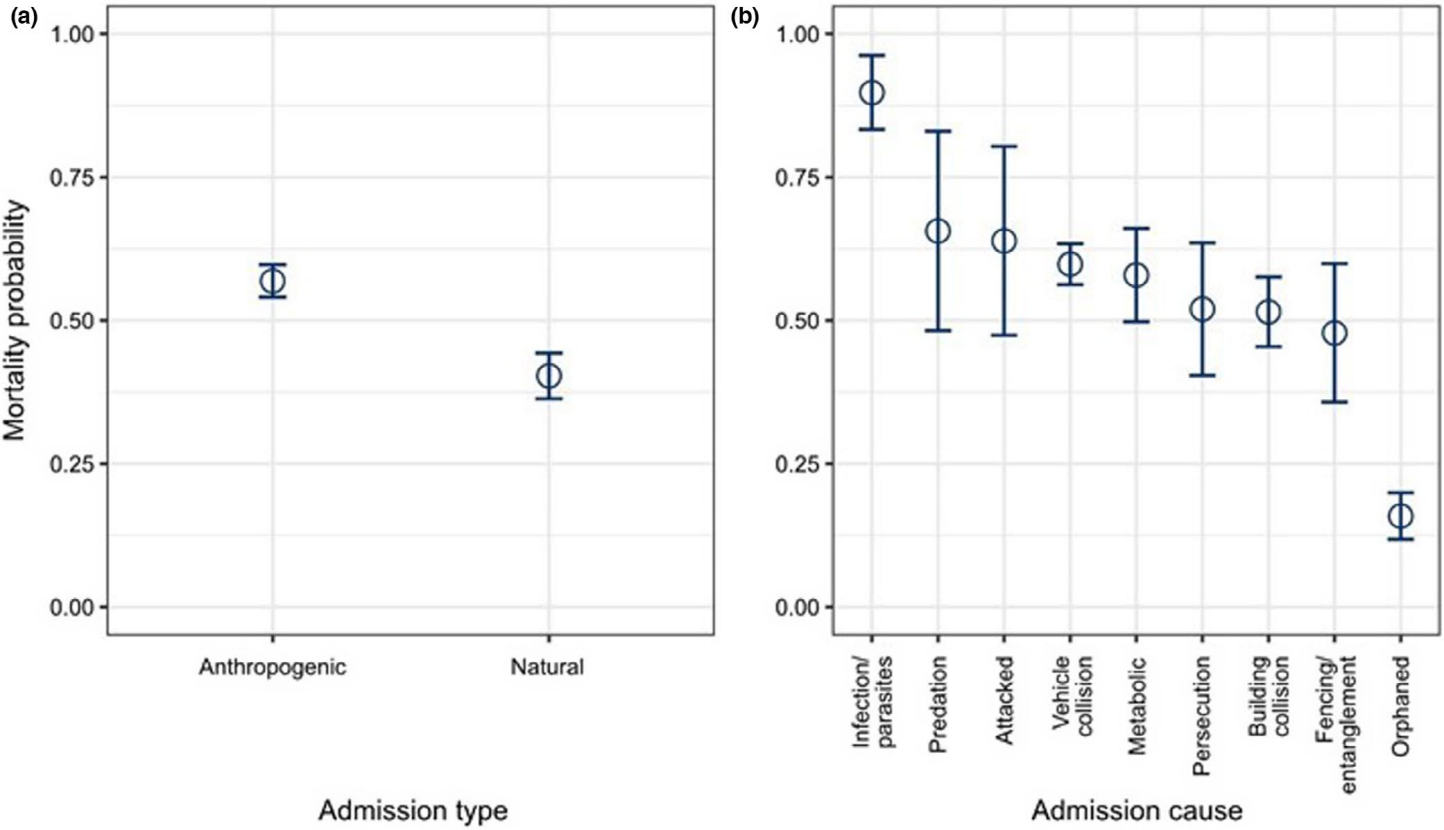
Differences in mortality probabilities for raptors admitted to four wildlife rehabilitation centers in England and Wales, between 2001 and 2019, in relation to identified (a) admission types and (b) admission causes. Data for “unknown” admission type not shown. Error bars represent 95% confidence intervals

Of all admissions, 60% resulted in the death or euthanasia of the bird, 39% resulted in the release of the bird, and just 1% of birds were kept in captivity post‐admission (Table 3). Those admitted for anthropogenic reasons had a significantly higher mortality rate (57%) than those admitted for natural reasons (40%) (*z*_1,1754_ = 6.483, *p* < .0001) (Figure 3a; Table 3; Appendix S3). Mortality probabilities differed among the most common admission causes (Figure 3b). Raptors admitted due to infection/parasites had a substantially higher mortality rate (90%) compared with other known admission causes, whereas orphaned birds had a significantly lower mortality rate (16%) than other known admission causes (Figure 3b; Table 3; Appendix S3).

**TABLE 3:** Overview of admission type, causes, and outcomes for all raptor admissions to four wildlife rehabilitation centers in England and Wales between 2001 and 2019

Type	Cause	Outcome			Total (%)
		Kept captive (%/cause)	Deceased/euthanized (%/cause)	Released (%/cause)	
Anthropogenic	Attacked by pet	0 (0)	23 (64)	13 (36)	36 (1)
	Building collision	1 (<1)	136 (52)	127 (48)	264 (8)
	Electrocution	0 (0)	11 (73)	4 (27)	15 (<1)
	Fencing/entanglement	1 (2)	33 (49)	35 (51)	69 (2)
	Habitat destruction	5 (21)	3 (16)	16 (67)	24 (1)
	Persecution	1 (1)	39 (53)	35 (47)	75 (2)
	Vehicle collision	1 (<1)	438 (60)	293 (40)	732 (22)
Total anthropogenic^a^		9 (<1)	683 (56)	523 (43)	1215 (37)
Natural	Infection/parasites	1 (1)	79 (91)	8 (9)	88 (3)
	Metabolic	0 (0)	84 (58)	61 (42)	145 (4)
	Orphaned	25 (8)	50 (17)	240 (76)	315 (10)
	Predation	0 (0)	21 (66)	11 (34)	32 (1)
Total natural^a^		26 (5)	234 (40)	320 (55)	580 (18)
Unknown	Trauma	3 (<1)	689 (81)	163 (19)	855 (26)
	Undetermined	5 (<1)	368 (57)	282 (43)	655 (20)
Total unknown^a^		8 (1)	1057 (70)	445 (29)	1510 (46)
Total admissions		43 (1)	1974 (60)	1288 (39)	3305 (100)

Note

Outcome proportions calculated per admission cause, total based on the total number of admissions.

^a^ Proportions calculated using total admission type values.

### 3.3 Trends over time in raptor admissions

**FIGURE 4:**
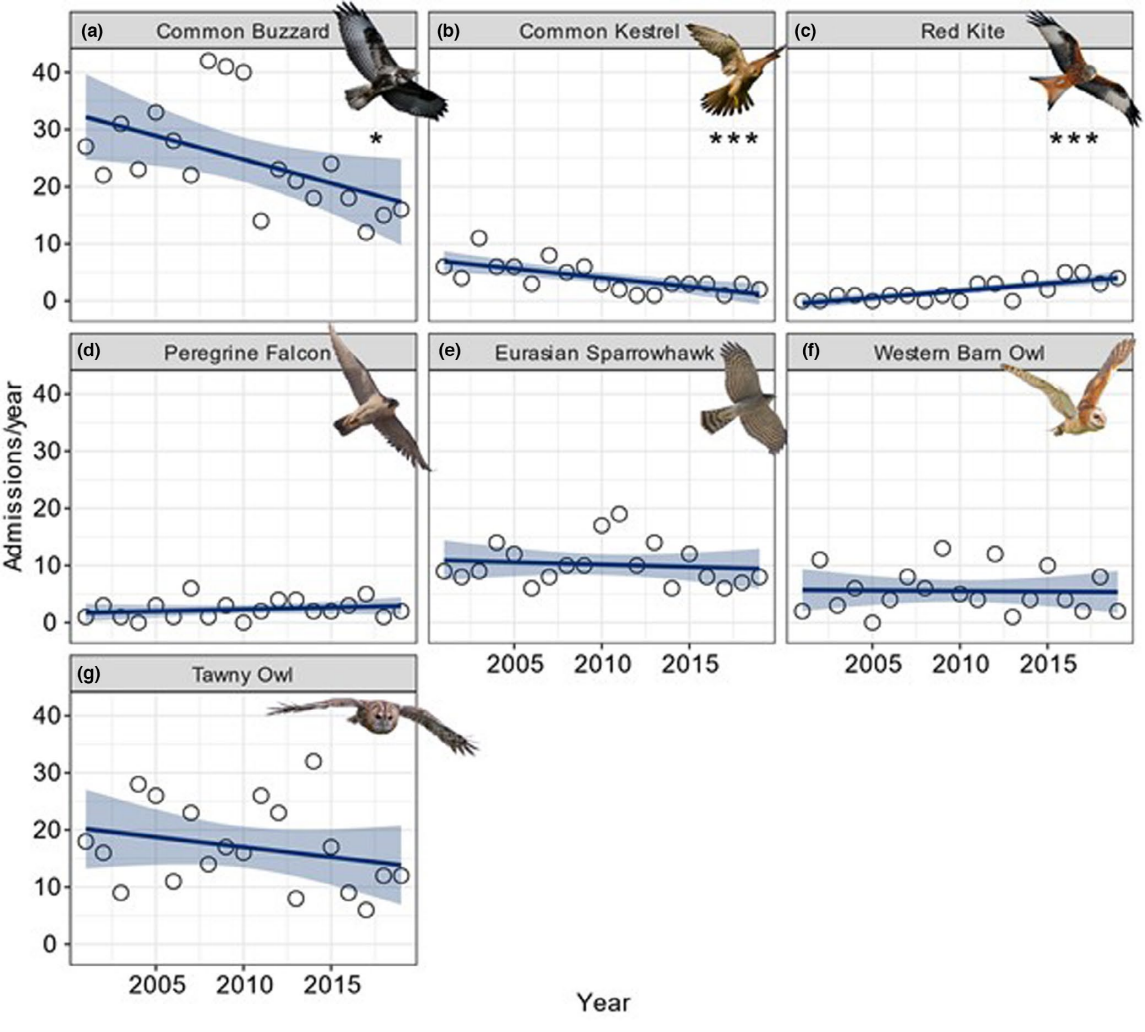
Trends over time for the seven most common raptor species admitted to Gower Bird Hospital between 2001 and 2019. (a) Common Buzzard (*Buteo buteo*; *N* = 470), (b) Common Kestrel (*Falco tinnunculus*; *N* = 77), (c) Red Kite (*Milvus milvus*; *N* = 34), (d) Peregrine Falcon (*Falco peregrinus*; *N* = 44), (e) Eurasian Sparrowhawk (*Accipiter nisus*; *N* = 193), (f) Western Barn Owl (*Tyto alba*; *N* = 105), and (g) Tawny Owl (*Strix aluco*; *N* = 323). Significant trends over time denoted by “***” = *p* < .001 and “*” = *p* < .05

Between 2001 and 2019, there was a notable decline in raptor admissions to Gower Bird Hospital when analyzing all admission types (*t*_1,17_ value = −2.164, *p* < .05). However, the relative proportion of known anthropogenic vs. natural admissions admitted to Gower Bird Hospital did not change over time (*z*_1,17_ = −1.554, *p* = .120). Over this period, there was a significant increase in the number of Red Kites admitted (*t*_1,17_ = 4.703, *p* < .001) (Figure 4). Conversely, there were significant declines in the number of Common Buzzards (*t*_1,17_ = −2.407, *p* < .05) and Common Kestrels admitted (*t*_1,17_ = −4.031, *p* < .001) (Figure 4; Appendix S4). We also saw a significant decline in the relative proportion of persecution and metabolic‐related admissions, and a significant increase in orphaned young birds, admitted to Gower Bird Hospital throughout the study period (Table 4).

**TABLE 4:** Trends over time in the relative proportion, per year, of admission causes for 1237 raptors admitted to Gower Bird Hospital between 2001 and 2019

Admission cause	*N*	Estimate ± SE	*t*	df	*p*
*Anthropogenic*					
Building collision	113	−0.012 ± 0.020	−0.607	18	.552
**Persecution**	**38**	**−0.074 ± 0.033**	**−2.258**	**–**	**<.05**
Vehicle collision	322	0.008 ± 0.014	0.563	–	.581
*Natural*					
Infection/parasites	41	0.042 ± 0.039	1.081	–	.295
**Metabolic**	**78**	**−0.072 ± 0.033**	**−2.149**	**–**	**<.05**
**Orphaned**	**97**	**0.066 ± 0.028**	**2.302**	**–**	**<.05**
*Unknown*					
Trauma	312	−0.011 ± 0.015	−0.766	–	.454
Undetermined	236	0.009 ± 0.020	0.466	–	.647

Note

Data were analyzed using a series of generalized linear models fitted with quasi‐Poisson error distributions to control for overdispersion. Only admission causes ≥30 were included. Bold = statistically significant causes.

Abbreviations: df, degrees of freedom; *N*, sample size; SE, standard error.

### 3.4 Effects of urbanization

From 3305 admissions, 1915 (58%) were geo‐referenced. For these geo‐referenced admissions, the mean percentage urban land cover within the 2‐km diameter buffers was 31 ± 28% (±SD) (Figure 1). We found no significant association between the proportion of urbanization for each geo‐referenced admission and the probability that the admission was caused by anthropogenic (*z*_1,1914_ = 0.940, *p* = .347), natural (*z*_1,1914_ = −1.085, *p* = .278) or unknown factors (*z*_1,1914_ = −0.118, *p* = .906). We did, however, find a significant positive association between urbanization and the probability of admission cause being building collisions, persecution, or unknown trauma (Table 5). In the least urbanized areas, the probability of admission being attributed to a building collision was only *c*. 7% but increased to *c*. 18% in the most urbanized areas. Likewise, persecution increased from *c*. 2.5% in the least urbanized areas to around 8% in the most urbanized areas. In contrast, vehicle collision admissions were negatively associated with urbanization, with a considerably higher probability of admissions being attributed to vehicle collisions in less urbanized areas—this was also the case for undetermined admission causes (Table 5). Urbanization was not associated with the probability of admission being attributed to any natural admission causes including infection/parasites, metabolic or orphaned young birds (Table 5).

**TABLE 5:** Effects of urbanization on causes of admission for raptors admitted to four wildlife rehabilitation centers in England and Wales between 2001 and 2019

Admission cause	*N*	Estimate ± SE	*z*	df	*p*
*Anthropogenic*					
**Building collision**	**136**	**0.011 ± 0.003**	**3.503**	**1109**	**<.001**
**Persecution**	**49**	**0.010 ± 0.005**	**2.047**	**–**	**<.05**
**Vehicle collision**	**503**	**−0.005 ± 0.002**	**−2.533**	**–**	**<.05**
*Natural*					
Infection/parasites	64	−0.001 ± 0.005	−0.223	–	.824
Metabolic	105	−0.005 ± 0.004	−1.464	–	.143
Orphaned	165	0.0005 ± 0.003	0.178	–	.859
*Unknown*					
**Trauma**	**456**	**0.004 ± 0.002**	**1.980**	**–**	**<.05**
**Undetermined**	**349**	**−0.005 ± 0.002**	**−2.529**	**–**	**<.05**

Note

Data were analyzed using a series of generalized linear mixed models fitted with binomial error distributions and “logit” link functions. Bold = statistically significant causes. Values computed using only geo‐referenced admissions with 2‐km diameter buffers.

### 3.5 Representation of raptor species

**FIGURE 5:**
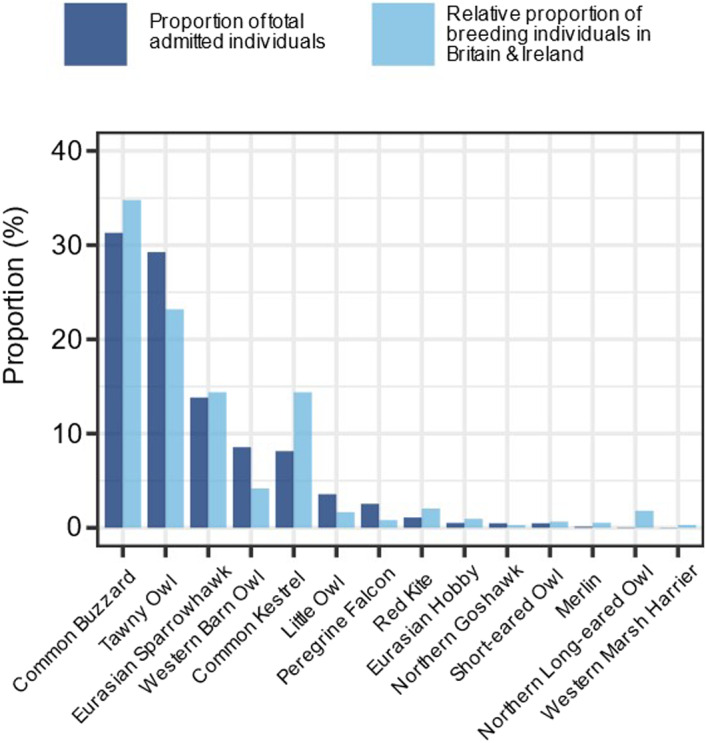
Proportion of total number of admitted individual raptors to four wildlife rehabilitation centers in England and Wales between 2001 and 2019, compared with the relative proportion of breeding individuals, per species, occurring in Britain and Ireland (data extracted from the BTO BirdFacts database https://www.bto.org/understanding‐birds/birdfacts; Robinson, 2005)

Compared with the relative proportion of breeding individuals in Britain and Ireland, some species were under‐ and over‐represented within our admissions data (Figure 5; Appendix S5). For example, Peregrine Falcons, Little Owls, and Western Barn Owls were over‐represented in our admissions data by 103%, 73%, and 69%, respectively (Figure 5; Appendix S5). Contrastingly, Northern Long‐eared Owls (*Asio otus*), Western Marsh Harriers (*Circus aeruginosus*), and Merlin (*Falco columbarius*) were under‐represented in our admissions data by 187%, 163%, and 126%, respectively (Figure 5; Appendix S5).

## 4 DISCUSSION

This study examines, over time, causes of morbidity and mortality for 14 raptors admitted to four wildlife rehabilitation centers in England and Wales and explores how urbanization affects causes of admission.

Similar to other studies, unknown trauma accounted for most raptor admissions to wildlife rehabilitation centers (WRC) (see Garcês et al., 2019; Mariacher et al., 2016; Rodríguez et al., 2010; Smith et al., 2018; Wendell et al., 2002). For example, Molina‐López et al. (2011) found that trauma accounted for 50% of raptor admissions to a WRC in Spain, with the cause of injury unascertainable for more than half of these. Trauma admissions were also most numerous (56%) in a study of 3212 raptor admissions to a WRC in New York State, USA (Hanson et al., 2021). In South Africa, an analysis of eight years of admissions data for 39 raptor species revealed that vehicle and building collisions were the most common cause of admission (Thompson et al., 2013), and another South African study found that 52% of all admissions for 33 raptor species were also due to collision‐related injuries (Maphalala et al., 2021). In our study, collision trauma (both building and vehicle collisions) comprised 56% of all identified admissions and a third of all admissions. In contrast, a 10‐year study conducted in Gran Canaria, Spain, found that 65% of raptor admissions were non‐trauma‐related, e.g., orphaned young birds, with trauma amounting to only around 35% of total admissions (Montesdeoca, Calabuig, Corbera, Rocha, et al., 2017).

Predominate causes of admission to WRC may vary by country. In Jordan, illegal possession and the transport of raptors was the most common admission cause to a single WRC center between 2017 and 2018, with trauma cases being the second most frequent admission cause (Al Zoubi et al., 2020). A recent study from the Czech Republic reported more than a third of all admissions of 12,923 Common Kestrels to 34 rehabilitation centers were due to nestlings/orphans (Lukesova et al., 2021). In this study, orphans accounted for 14% of total kestrel admissions and together with vehicle collisions were the most frequent admission cause for this species.

In our study, nearly 60% of admitted birds either died or were euthanised. Admissions due to anthropogenic causes had a higher mortality rate (57%) than natural causes (40%), and our more refined analysis suggested that infection/parasite admissions were associated with the highest mortality rates (90%), whereas orphaned birds were associated with the lowest mortality rate (16%). Raptors admitted due to being orphaned tend to have higher survival probabilities than those admitted for other reasons, as evidenced by existing studies (Hanson et al., 2021; Lukesova et al., 2021 [see “Nestlings” and “Incubation” in Table 3]).

### 4.1 Influence of urbanization on identified causes of admission

The level of urbanization was significantly associated with certain admission causes, with building collisions, persecution, and unknown trauma admissions more likely to occur in more urbanized areas, but with vehicle collisions more likely in rural areas. Compared with diurnal species, nocturnal species are more susceptible to blinding by vehicle headlights (Bullock et al., 2011; Thompson et al., 2013). Collisions between Tawny Owls and vehicles have been shown to be more common on roads surrounded by increased tree density (Gomes et al., 2009) where connectivity between territories is higher (Gagné et al., 2015; Santos et al., 2013), i.e., more rural areas, and may explain why vehicle collisions were the most frequent identified admission cause for Tawny Owls in our study. Common Buzzards were the most numerous diurnal species hit by vehicles; the species is less able to adapt to urban habitats (Palomino & Carrascal, 2007) and is also a frequent scavenger of roadkill carcasses in rural areas (Schwartz et al., 2018; Young et al., 2014), which may further explain the increase in vehicle collisions in more rural areas. Vehicle collisions were also the most common admission cause for Western Barn Owls totalling 40% of admissions and were also the most likely cause of death for the species in another study conducted in Britain between 1963–1996 (Newton et al., 1997).

Building collisions were more likely to occur in urban areas with the Eurasian Sparrowhawk being the most frequent species admitted for this reason. This species is an urban adapter often breeding in these environments (Thornton et al., 2017) employing a high‐speed attack strategy when hunting avian prey (Newton, 1986). Important causes of mortality have been attributed to collision‐based trauma particularly with windows (Newton et al., 1999). A study by Kelly and Bland (2006) analyzed 202 admissions of Eurasian Sparrowhawk to a WRC in England, 32% of admissions were due to collisions, i.e., vehicle and building/window collisions, which is an identical percentage to our findings for this species admitted to four WRC, suggesting that collision‐based injuries (and/or death) are relatively common for the species in England and Wales (Newton et al., 1999).

Recently, Crespo et al. (2021) found a positive relationship between the number of human inhabitants and avian gunshot admissions in the Valencian region of Spain, the majority of casualties being raptors. We did not explore the effects of human population densities on admission causes; however, we found that persecution admissions (i.e., gunshots, poisoning, and traps/snares) increased in urban areas. Assuming that human population densities correlate with urban land cover, our results are in line with those of Crespo et al. (2021). Despite this, in Britain, it is well‐documented that human‐raptor conflict often occurs in rural areas such as grouse moors (Melling et al., 2018; Murgatroyd et al., 2019; Newton, 2021; Thirgood et al., 2001), although there is no active grouse moor management within our study area, and this pattern might well change if these issues were explored at a larger scale incorporating a wider range of habitat types.

The lack of randomization (Molina‐López et al., 2011), restricted geographic study area, and small sample sizes for less abundant species (e.g., a single admission for the Western Marsh Harrier and no admissions of species such as the Hen Harrier (*Circus cyaneus*) despite overlap with the species’ distribution in Wales) further limit our ability to explore trends in causes of injury and death for all raptor species occurring throughout England and Wales. Peregrine Falcons were over‐represented in our admissions data by 103%. This may be due to recent estimates suggesting that the species’ population size has increased in lowland parts of England along with the overall UK population (Wilson et al., 2018) and/or may be due to the species’ well‐known use of urban habitats (Kettel et al., 2019), subsequently increasing the chance of members of the public encountering injured falcons. Conversely, Northern Long‐eared Owls were under‐represented in our admissions data by 187%, totalling just two admissions over the study period. In Britain and Ireland, the species’ estimated breeding population size (*c*. 7800 individuals) is larger than that of other species that were more numerous within our admissions data, e.g., Little Owl (118 admissions; *c*. 7200 breeding individuals), Northern Goshawk (16 admissions; *c*. 1240 breeding individuals) and Peregrine Falcon (84 admissions; *c*. 3500 breeding individuals). Northern Long‐eared Owls are nocturnal and secretive (Petty et al., 2003), preferring to use habitats away from human disturbance (Martínez & Zuberogoitia, 2004), which may partially explain the low numbers observed in our admissions data.

Admission cause in most cases was based upon details from the finder of the bird (usually a member of the public) and an initial assessment by a trained wildlife carer. A veterinary professional (veterinary surgeon or registered veterinary nurse) was usually not involved at this stage, so a definitive clinical diagnosis was not made. The centers involved, however, all have very experienced and well‐trained staff, with the ability to make a good initial assessment of the bird. However, identification accuracy between WRC and trained wildlife carers is unlikely to be equal, which should be considered when making inferences from these data.

For 77% of admissions, sex was not determined, constraining our ability to compare admission causes between the sexes. However, the majority of admitted birds were able to be assigned to a broad age category allowing for age‐related demographic comparisons. Nevertheless, 60% of admissions were of adult birds, which support results from WRC in the USA (Hernandez et al., 2018) and Greece (Komnenou et al., 2005). The remaining 40% of admissions comprised juvenile birds and similar patterns have been observed elsewhere; for example, 42% of Northern Long‐eared Owl admissions (Italy; Mariacher et al., 2016) and 32% of all raptor admissions (Spain; Molina‐López et al., 2011) being juveniles.

Relative to anthropogenic admissions, natural admissions are likely to be under‐represented in our data due to the majority going unreported (Newton, 2002; Real et al., 2001). The reliance of reports from members of the public means that there is a likely bias towards anthropogenic admission causes. Building and vehicle collisions are more likely to be reported by members of the public by chance than persecution, i.e., illegal activities such as poisoning, gunshot, and trap/snare events. Our data may also include a survivability bias with members of the public more likely to report injured birds that are still alive than those that have already died, inhibiting reliable injury and death estimates at local raptor population levels.

Alternative monitoring methods such as satellite telemetry are more reliable sources for capturing illegal wildlife crimes, as demonstrated by Murgatroyd et al. (2019) who examined patterns of Hen Harrier disappearances over grouse moors in northern England as a result of suspected illegal killing. In addition, Panter et al. (2021) used satellite telemetry to estimate survival in wintering Red Kites in south‐western Europe and Oppel et al. (2021) coupled satellite telemetry and on‐the‐ground surveys to explore Egyptian Vulture (*Neophron percnopterus*) mortalities along their migratory routes. However, using such technology is often costly and requires specialist skills. Analysis of admissions data is cost effective and requires little investment other than time, and many WRC often keep records of wildlife admissions for their own purposes as demonstrated in this study.

### 4.2 Implications

Admissions data from WRC have the potential to form important baseline data guiding conservation activities. For example, gunshot admissions data from Greece have been used to advise governmental agencies responsible for hunting regulations (Mazaris et al., 2008) and seasonal cumulative indices have been calculated to explore the potential ecological impacts on local raptor populations in Spain (Molina‐López et al., 2011). Some 39% of raptors were released back into the wild following treatment; however, the release does not equate to successful reintroduction back into breeding populations. Post‐release monitoring of individuals, for example, via identification of individuals using leg bands and coupled with field surveys, is strongly encouraged. This provides additional conservation value to admissions data and also allows for post‐release welfare checks to be made on the bird.

Building and vehicle collisions posed the highest identified risk to raptors in our study area. Increased traffic densities and vehicle speeds have been shown to increase bird‐vehicle collision mortalities (Erritzoe et al., 2003). Identification of vehicle collision hotspots along road networks is recommended, and predictive modeling has been applied at the landscape and local scale to improve road safety (Malo et al., 2004). Window decals have successfully reduced average monthly bird‐window collisions by 84% (Ocampo‐Peñuela et al., 2016). Application of collision prevention decals to the exterior surface of windows (Klem & Saenger, 2012), or tinting of windows (Erickson et al., 2005), are viable solutions to prevent bird‐building collisions and citizen science can assist with community‐level implementations.

Transformation of natural habitats into human‐modified environments has been shown to negatively affect raptor communities, resulting in lower abundances, species richness, and diversity (Carrete et al., 2009). Despite this, some raptor species have shown resilience and even proliferation in urban environments (Cooke et al., 2018; Kettel et al., 2019; Panter et al., 2020). For example, Sumasgutner et al. (2020) found that breeding Peregrine Falcon pairs were more likely to breed and bred earlier in more urbanized areas, compared with their more rural conspecifics, but breeding success may be compromised in more urban areas for some species, e.g., Common Kestrels (Kettel et al., 2018).

Many threats persist for raptors in England and Wales, however, have not changed substantially over the past two decades. Our findings provide baseline data on the causes of morbidity and mortality of raptors throughout our study area. Threats associated with urban areas, such as building collisions, may increase over time in line with human population growth and subsequent urban expansion. There is potential for future studies to build on our results in an applied context, for example, investigating the financial costs of vehicle damage as a result of vehicle‐wildlife collisions.

## CONFLICT OF INTEREST

The authors declare no conflict of interest.

## AUTHOR CONTRIBUTIONS

**Connor T. Panter:**Conceptualization (lead); Data curation (lead); Formal analysis (equal); Investigation (equal); Methodology (equal); Project administration (lead); Visualization (lead); Writing – original draft (lead); Writing – review & editing (equal). **Simon Allen:** Resources (equal); Writing – review & editing (equal). **Nikki Backhouse:** Resources (equal). **Elizabeth Mullineaux:** Investigation (equal); Resources (equal); Writing – review & editing (equal). **Carole‐Ann Rose:** Resources (equal); Writing – review & editing (equal). **Arjun Amar:** Formal analysis (equal); Investigation (equal); Methodology (equal); Supervision (lead); Validation (equal); Writing – review & editing (lead).

## Supporting information

Supplementary Material

## Data Availability

Data associated with this study will be available via https://github.com/ConnorPanter/Panter‐et‐al‐2022‐Ecology‐and‐Evolution.
