# Supplementary material for: Causes, temporal trends, and the effects of urbanization on admissions of wild raptors to rehabilitation centers in England and Wales
Source: Ecol Evol. 2022 Apr 20;12(4):e8856. doi: 10.1002/ece3.8856 (PMC9020437; doi:10.1002/ece3.8856)
Supplement: Supplementary file 1 — Supplementary Material [file ECE3-12-e8856-s001.docx]

**APPENDICES**

**Appendix S1.** Admission type, cause, code and descriptions for 3305 admission records of raptors admitted to four wildlife rehabilitation centres in the United Kingdom between 2001-2019.

| Admission type | Admission cause | Code | Description |
| --- | --- | --- | --- |
| Anthropogenic (7) | Attacked by pet | ‘attack’ | Admissions where finder observed casualty being attacked by domesticated animal (e.g., cat or dog) |
|  | Building collision | ‘build’ | Category also includes collisions with manmade structures, birds falling down chimneys, trapped down wells or drains and trapped in sheds or greenhouses |
|  | Electrocution | ‘elec’ | Admissions where casualty has been electrocuted on powerlines |
|  | Fencing/entanglement | ‘fence’ | Category also includes casualties caught in netting over ponds and cattle grids |
|  | Habitat destruction | ‘habitat’ | Admissions where finder reports casualties being disturbed in the nest or disturbance to breeding adult pairs by felling trees, machinery and vegetation clearance |
|  | Persecution | ‘pers’ | Category includes direct and indirect persecution also includes gunshot victims, poisoning and traps or snares |
|  | Vehicle collision | ‘veh’ | Category also includes stunned casualties found on roads |
| Natural (4) | Infection/parasites | ‘infect’ | Category includes capsulitis diagnosed with infections, parasites, abnormal growths and tumours |
|  | Metabolic | ‘metab’ | Admissions where casualty has signs of emaciation, starvation and heat exhaustion |
|  | Orphaned | ‘orph’ | Admissions where finder reports casualties outside of the natal nest |
|  | Predation | ‘pred’ | Admissions where finder reports physical injury/wounds to causality but not as a result of domesticated animals |
| Unknown (2) | Trauma | ‘trauma’ | Category includes all casualties with physical injuries/wounds or those in shock where the exact cause could not be ascertained |
|  | Undetermined | ‘undet’ | Category includes casualties without physical injuries/wounds, not in shock and where exact cause could not be ascertained |

**Appendix S2.** An overview of models used to explore trends over time and effects of urbanisation on raptor admissions to four wildlife rehabilitation centres (WRC) in England and Wales between 2001-2019. GLM = Generalized Linear Model; GLMM = Generalized Linear Mixed Model; GBH = Gower Bird Hospital only.

| Model type | Terms | | | Error distribution | Link function | Data set | Data included (admission types) | | |
| --- | --- | --- | --- | --- | --- | --- | --- | --- | --- |
|  | Response | Explanatory | Random |  |  |  | Anthropogenic | Natural | Unknown |
| GLM | fate (binary) | admission type/cause | - | binomial | logit | All WRC | 🗸 | 🗸 |  |
| GLM | total admission counts | year | - | quasi-Poisson | - | GBH | 🗸 | 🗸 | 🗸 |
| cbind GLM | admission counts per type (anthropogenic vs. natural) | year | - | binomial | - | GBH | 🗸 | 🗸 |  |
| GLM | admission counts per species | year | - | quasi-Poisson | - | GBH | 🗸 | 🗸 | 🗸 |
| GLM | relative proportion of admission counts by per cause | year | - | quasi-Poisson | - | GBH | 🗸 | 🗸 | 🗸 |
| GLMM | anthropogenic (binary) | % urban land cover | centre ID | binomial | logit | All WRC | 🗸 |  |  |
| GLMM | natural (binary) | % urban land cover | centre ID | binomial | logit | All WRC |  | 🗸 |  |
| GLMM | unknown (binary) | % urban land cover | centre ID | binomial | logit | All WRC |  |  | 🗸 |
| GLMM | cause (binary) | % urban land cover | centre ID | binomial | logit | All WRC | 🗸 | 🗸 | 🗸 |

**Appendix S3.** Pairwise comparisons of mortality probabilities for raptors admitted to four wildlife rehabilitation centres in England and Wales between 2001-2019, presented by identified admission types (above dashed line) and admission causes (below dashed lines). Analyses conducted using a series of Generalized Linear Models with binomial error distributions and ‘logit’ link functions. **Bold** = statistically significant comparisons, SE = standard error.

| Contrast | Estimate ± SE | *z* | *P* |
| --- | --- | --- | --- |
| **anthropogenic - natural** | **0.668 ± 0.103** | **6.483** | **< 0.0001** |
| attacked - building collision | 0.509 ± 0.369 | 1.385 | 0.904 |
| attacked - fencing/entanglement | 0.657 ± 0.422 | 1.556 | 0.828 |
| **attacked - infection/parasites** | **-1.601 ± 0.494** | **-3.241** | **< 0.05** |
| attacked - metabolic | 0.250 ± 0.386 | 0.650 | 0.999 |
| **attacked - orphaned** | **2.238 ± 0.380** | **5.895** | **< 0.0001** |
| attacked - persecution | 0.490 ± 0.417 | 1.176 | 0.962 |
| attacked - predation | -0.076 ± 0.509 | -0.150 | 1.000 |
| attacked - vehicle collision | 0.171 ± 0.355 | 0.484 | 1.000 |
| building collision - fencing/entanglement | 0.147 ± 0.271 | 0.546 | 1.000 |
| **building collision - infection/parasites** | **-2.116 ± 0.373** | **-5.665** | **< 0.0001** |
| building collision - metabolic | -0.259 ± 0.208 | -1.244 | 0.947 |
| **building collision - orphaned** | **1.728 ± 0.197** | **8.759** | **< 0.0001** |
| building collision - persecution | -0.019 ± 0.262 | -0.074 | 1.000 |
| building collision - predation | -0.586 ± 0.392 | -1.495 | 0.858 |
| building collision - vehicle collision | -0.338 ± 0.144 | -2.341 | 0.317 |
| **fencing/entanglement - infection/parasites** | **-2.259 ± 0.426** | **-5.298** | **< 0.0001** |
| fencing/entanglement - metabolic | -0.407 ± 0.294 | -1.385 | 0.904 |
| **fencing/entanglement - orphaned** | **1.580 ± 0.286** | **5.525** | **< 0.0001** |
| fencing/entanglement - persecution | -0.167 ± 0.334 | -0.500 | 1.000 |
| fencing/entanglement - predation | -0.733 ± 0.443 | -1.655 | 0.774 |
| fencing/entanglement - vehicle collision | -0.485 ± 0.253 | -1.923 | 0.597 |
| **infection/parasites - metabolic** | **1.852 ± 0.390** | **4.750** | **< 0.0001** |
| **infection/parasites - orphaned** | **3.839 ± 0.384** | **9.997** | **< 0.0001** |
| **infection/parasites - persecution** | **2.092 ± 0.421** | **4.971** | **< 0.0001** |
| infection/parasites - predation | 1.525 ± 0.512 | 2.979 | 0.071 |
| **infection/parasites - vehicle collision** | **1.773 ± 0.360** | **4.930** | **< 0.0001** |
| **metabolic - orphaned** | **1.987 ± 0.228** | **8.710** | **< 0.0001** |
| metabolic - persecution | 0.239 ± 0.286 | 0.839 | 0.996 |
| metabolic - predation | -0.326 ± 0.408 | -0.800 | 0.997 |
| metabolic - vehicle collision | -0.078 ± 0.184 | -0.427 | 1.000 |
| **orphaned - persecution** | **-1.747 ± 0.278** | **-6.291** | **< 0.0001** |
| **orphaned - predation** | **-2.314 ± 0.403** | **-5.745** | **< 0.0001** |
| **orphaned - vehicle collision** | **-2.066 ± 0.172** | **-12.039** | **< 0.0001** |
| persecution - predation | -0.566 ± 0.438 | -1.293 | 0.934 |
| persecution - vehicle collision | -0.318 ± 0.243 | -1.310 | 0.929 |
| predation - vehicle collision | 0.248 ± 0.380 | 0.653 | 0.999 |

**Appendix S4**. Parameter estimates from the Generalized Linear Models, fitted with a quasi-Poisson error distribution to account for overdispersion, examining trends over time for the seven most common species admitted to Gower Bird Hospital between 2001-2019. **Bold** = statistically significant causes. N = number of admissions, SE = standard error, df = degrees of freedom.

| Species | N | Estimate ± SE | *t* | df | *P* |
| --- | --- | --- | --- | --- | --- |
| **Common Buzzard (*Buteo buteo*)** | **470** | **-0.034 ± 0.014** | **-2.407** | **17** | **< 0.05** |
| Tawny Owl (*Strix aluco*) | 323 | -0.021 ± 0.019 | -1.112 | - | 0.282 |
| Eurasian Sparrowhawk (*Accipiter nisus*) | 193 | -0.008 ± 0.016 | -0.521 | - | 0.609 |
| Western Barn Owl (*Tyto alba*) | 105 | -0.003 ± 0.029 | -0.117 | - | 0.908 |
| **Common Kestrel (*Falco tinnunuculus*)** | **77** | **-0.081 ± 0.020** | **-4.031** | **-** | **< 0.001** |
| Peregrine Falcon (*Falco peregrinus*) | 44 | 0.028 ± 0.030 | 0.926 | - | 0.368 |
| **Red Kite (*Milvus milvus*)** | **34** | **0.155 ± 0.033** | **4.703** | **-** | **< 0.001** |

**Appendix S5.** Percentage difference between the relative proportion of breeding individuals in Britain & Ireland, and the proportion of individuals, per species, admitted to four wildlife rehabilitation centres in England and Wales between 2001-2019. *Data derived from the British Trust for Ornithology’s BirdFacts database (Robinson 2005; <https://www.bto.org/understanding-birds/birdfacts>).

| Species | Number of breeding individuals in Britain & Ireland* | Proportion breeding individuals in Britain & Ireland | Number of admitted individuals | Proportion of total admitted individuals | % Difference (breeding individuals vs. admitted individuals) |
| --- | --- | --- | --- | --- | --- |
| Peregrine Falcon (*Falco peregrinus*) | 3500 | 0.8 | 84 | 2.5 | 103.2 |
| Little Owl (*Athene noctua*) | 7200 | 1.7 | 118 | 3.6 | 72.5 |
| Western Barn Owl (*Tyto alba*) | 18000 | 4.2 | 283 | 8.6 | 68.9 |
| Northern Goshawk (*Accipiter gentilis*) | 1240 | 0.3 | 16 | 0.5 | 50.9 |
| Tawny Owl (*Strix aluco*) | 100000 | 23.2 | 967 | 29.3 | 23.1 |
| Eurasian Sparrowhawk (*Accipiter nisus*) | 62000 | 14.4 | 457 | 13.8 | -3.9 |
| Common Buzzard (*Buteo buteo*) | 150000 | 34.8 | 1035 | 31.3 | -10.5 |
| Short-eared Owl (*Asio flammeus*) | 2820 | 0.7 | 16 | 0.5 | -29.9 |
| Common Kestrel (*Falco tinnunculus*) | 62000 | 14.4 | 269 | 8.1 | -55.5 |
| Eurasian Hobby (*Falco subbuteo*) | 4100 | 1.0 | 17 | 0.5 | -59.6 |
| Red Kite (*Milvus milvus*) | 8800 | 2.0 | 36 | 1.1 | -60.8 |
| Merlin (*Falco columbarius*) | 2300 | 0.5 | 4 | 0.1 | -126.0 |
| Western Marsh Harrier (*Circus aeruginosus*) | 1285 | 0.3 | 1 | 0.0 | -163.1 |
| Northern Long-eared Owl (*Asio otus*) | 7800 | 1.8 | 2 | 0.1 | -187.1 |
